# Sustainable Co-Extraction of Starch and Tannins from *Quercus robur* Acorns Using Pulsed Electric Field Technology and Evaluation of Starch Gelatinization Properties

**DOI:** 10.3390/gels12090756

**Published:** 2026-08-24

**Authors:** Luís M. G. Castro, Carlos A. Pinto, Sérgio C. Sousa, Elisabete M. C. Alexandre, Jorge A. Saraiva, Manuela Pintado

**Affiliations:** 1 CBQF—Centro de Biotecnologia e Química Fina—Laboratório Associado, Escola Superior de Biotecnologia, Universidade Católica Portuguesa, Rua Diogo Botelho 1327, 4169-005 Porto, Portugal; 2 LAQV-REQUIMTE—Laboratório Associado, Department of Chemistry, University of Aveiro, 3810-193 Aveiro, Portugal

**Keywords:** *Quercus* spp., pulsed electric field, tannin recovery, starch extraction, gelatinization properties

## Abstract

Pulsed electric field (PEF) technology was used as a green extraction method for the simultaneous recovery of starch and polyphenols from acorns using water as extraction solvent, and the gelatinization properties of the extracted starch were compared with those of a starch obtained via alkaline extraction. Results showed that the electric field intensity had a significant negative quadratic effect on the antioxidant activity and hydrolyzable tannin content, whereas it had a positive quadratic effect on the starch yields. Time had no significant effect on the starch yields but had a negative quadratic effect on the phenolic and hydrolyzable tannin contents and antioxidant activity. The extraction condition that allowed the highest starch and polyphenol contents with the greatest antioxidant activity was 0.1 kV/cm for 63.3 µs, with a desirability of 87%. In comparison to alkaline extraction, PEF increased tannin extraction by 11-fold, highlighting its potential to produce tannin-rich extracts suitable for leather manufacture. Although the starch yield was 30% lower than that obtained via alkaline extraction, the PEF-isolated starch is a cleaner and safer ingredient for human consumption. The alkaline starch had a significantly higher solubility than the PEF-isolated starch, but both had similar swelling power.

## 1. Introduction

The *Quercus* spp. comprises over 500 species of evergreen or deciduous trees that produce a small fruit named an acorn [1,2]. Acorns are abundantly produced in Portugal and are very rich in carbohydrates, mainly starch. However, more than 50% of the annual production remains underused, with most of it being used as animal feed [1,3,4,5,6,7]. Since starch is an important macronutrient that represents 50 to 70% of the energy in the human diet, acorns represent a promising and largely unexplored starch source [8].

In addition to starch, acorns also contain significant amounts of tannins. Although these compounds are often regarded as anti-nutritional compounds, they are a valuable natural resource for industrial applications. Tannins can be used in leather manufacturing as a sustainable alternative to chromium salts, which raise environmental and health concerns [9,10,11,12,13]. Thus, exploiting acorns as a starch and tannin source offers an attractive strategy to increase the economic value of this underused resource while supporting more sustainable and circular biorefinery approaches. To maximize the value of acorns, both starch and tannins should be recovered through an integrated extraction process, but starch granules must be extracted without significant modifications to their structural integrity to be economically viable [14]. According to the literature, low-alkaline extraction with three sieves is considered the most effective method to isolate starch, providing high yields, purity, and preservation of granular morphology [15]. Nevertheless, basic alkaline-treated starches are yet to be approved as an independent, labeled food additive in the European Union [16]. Thus, it is important to create clean-label starches and extract the phenolic compounds using environmentally friendlier solvents and technologies [17].

Pulsed electric field (PEF) is a processing technique that is governed by the electro-pulsation phenomenon, i.e., the exposure of cells to short, high-voltage electric pulses. This process increases the permeability of the cell membranes through the creation of transmembrane pores, thereby facilitating the release of intracellular components [18]. PEF has been extensively used as an extraction-assisted technique for a variety of matrices, with overall results demonstrating higher extraction yields, enhanced selectivity, and the capacity to preserve thermolabile compounds. However, aside from its modification, no research was found on its suitability for extracting starch [18,19]. Depending on the processing conditions, PEF can increase starch granule size and size distribution, induce gelatinization, destroy crystalline structures, lower gelatinization temperatures and enthalpies, improve in vitro digestibility, and reduce peak and breakdown viscosities of starches when higher electric field intensities are applied. At lower electric field intensities, the crystalline structures of starches are retained while increasing in vitro digestibility. Gelatinization temperatures and enthalpies can rise or fall depending on the intensity; however, the pasting characteristics are often less adversely impacted than at higher intensities [18]. Thus, PEF offers the possibility to produce starches with tailored functional properties.

The objectives of this study are to (1) evaluate the effects of electric field intensity and extraction time on the simultaneous extraction of starch and phenolics from acorn cotyledons; (2) optimize the extraction process using multi-response processes to attain maximum yields; (3) compare the morphology of starch granules obtained under PEF optimum conditions with those and obtained via alkaline extraction; and (4) evaluate and compare the gelatinization properties (solubility and swelling power) of acorn starch obtained by both methodologies.

## 2. Results and Discussion

### 2.1. Effect of Electric Field Intensity

#### 2.1.1. Phenolic and Tannin Contents

**Figure 1:**
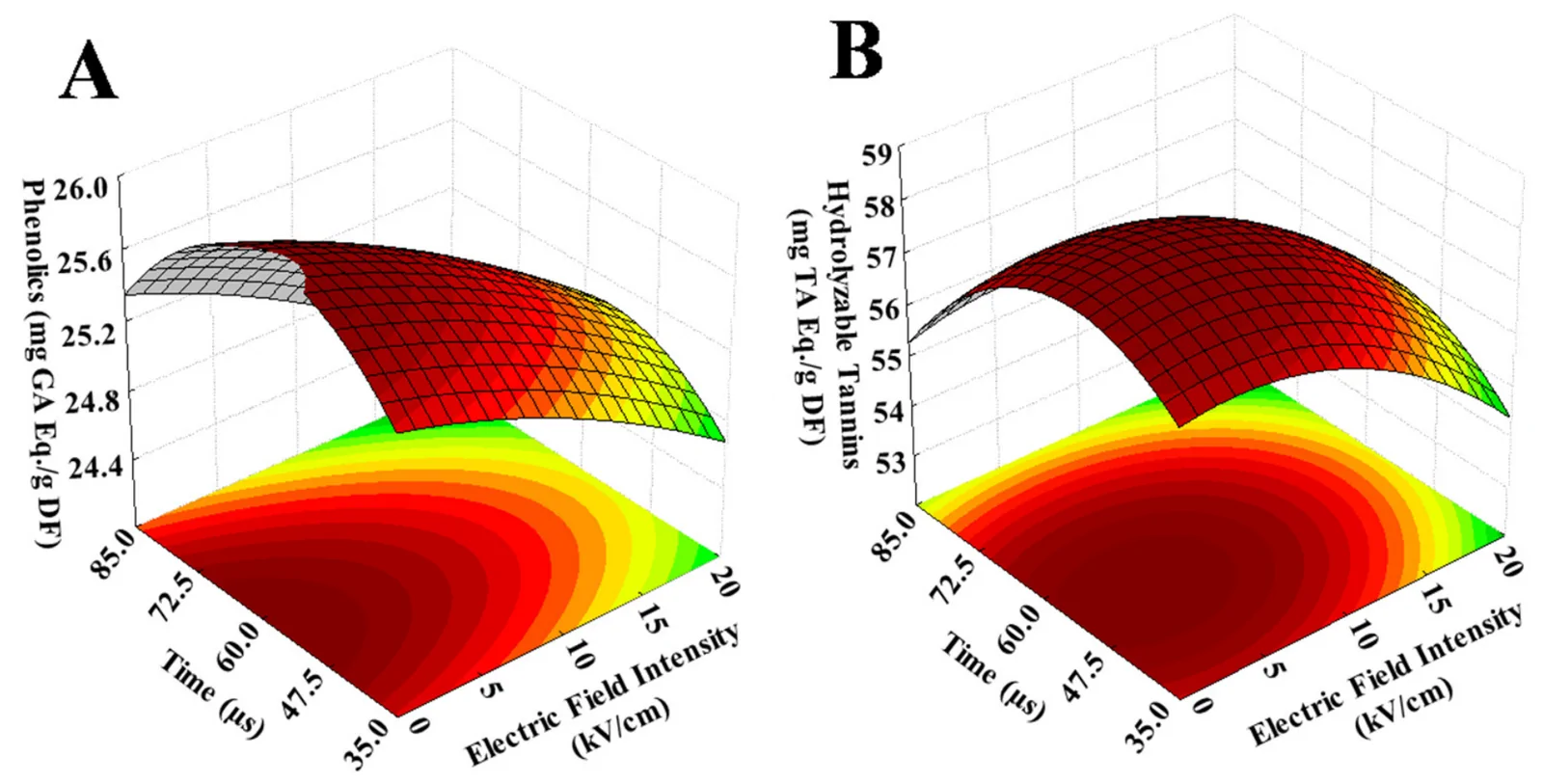
Response surface plots of the phenolics (**A**) and hydrolyzable tannins (**B**) from *Q. robur* acorns obtained by pulsed electric field extraction.

The electric field intensity did not have a significant effect on the extraction of phenolics from *Q. robur* (Figure 1A and Table 1). This result is consistent with previous observations for brewer’s spent grains and tinned peach by-products [20,21]. In contrast, other studies have reported that electric field intensity had a negative quadratic effect on the extraction of phenolics from another brewer’s spent grains [22], a positive quadratic effect on the extraction of phenolics from cocoa bean shells, coffee silverskin, and defatted flaxseed cake [23,24], and a positive linear response for cinnamon and onions [25,26].

For hydrolyzable tannins, the electric field intensity had a negative quadratic effect (Table 1). This behavior indicates that moderate electric field intensities favored tannin extraction, whereas lower or higher intensities reduced its extraction (Figure 1B). Previous authors also reported a decrease in the phenolic content from tomato at higher field intensities [27]. It is thought that such high electric field intensities might have damaged cell structure due to permanent losses of membrane permeability and have led to tannin degradation [28]. Furthermore, the polarization effect of starch molecules and other structures may have enhanced electrostatic interactions with tannins, subduing their diffusion into the extraction medium [29].

Condensed tannins were found in non-quantifiable levels, which may have been due to their reduced solubility in water [30].

#### 2.1.2. Antioxidant Activity

**Figure 2:**
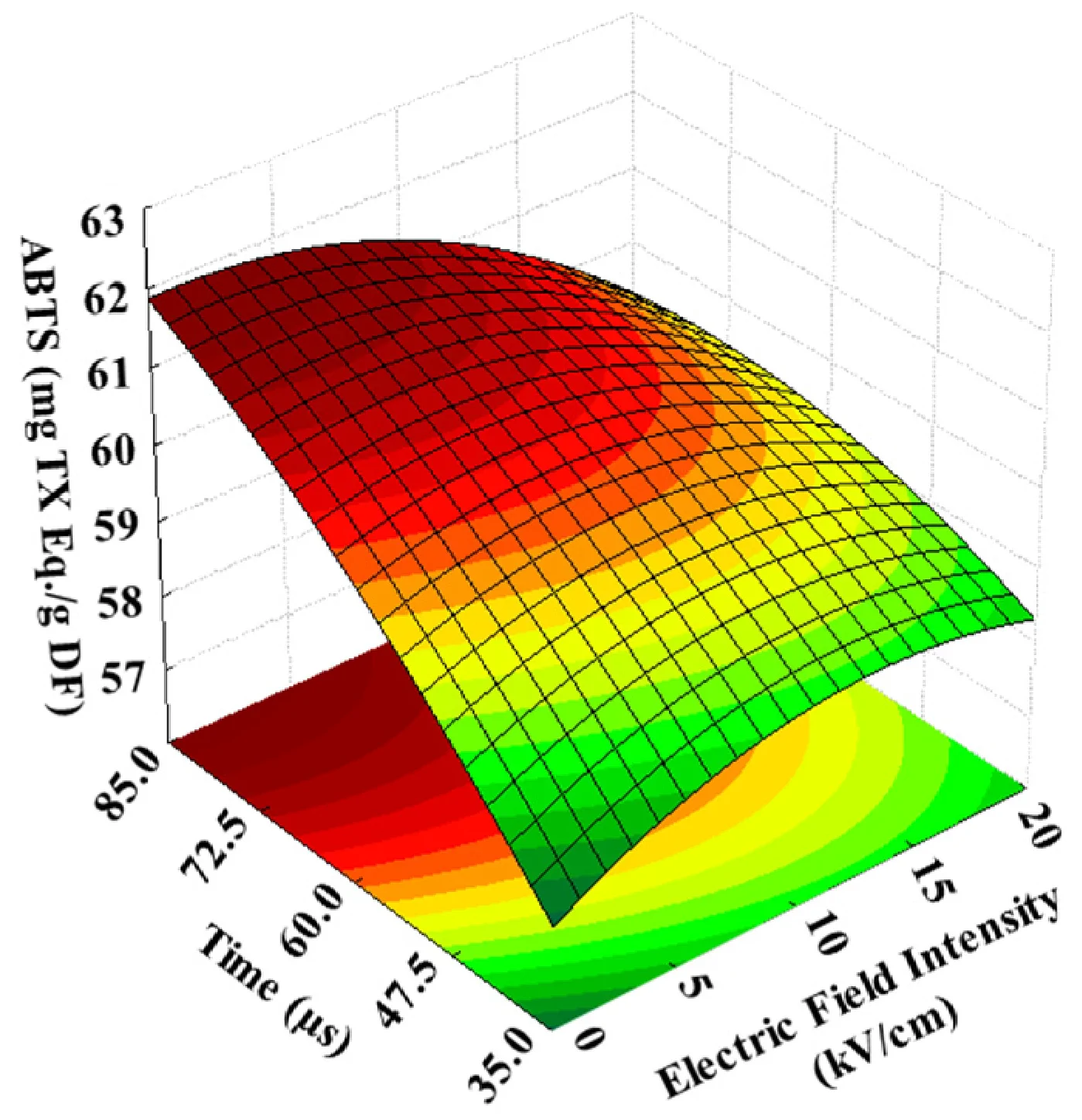
Response surface plots of the ABTS antioxidant activity from *Q. robur* acorns obtained by pulsed electric field extraction.

The electric field intensity had a significant negative quadratic effect on the antioxidant activity, indicating that the antioxidant activity first increases with the electric field intensity but then decreases when higher electric field intensities are applied (Table 1 and Figure 2). This result is consistent with that reported for tomatoes [27]. Antioxidant activity decreases at higher intensities, indicating that the stability of the antioxidant compounds may have been compromised.

#### 2.1.3. Starch Content

**Figure 3:**
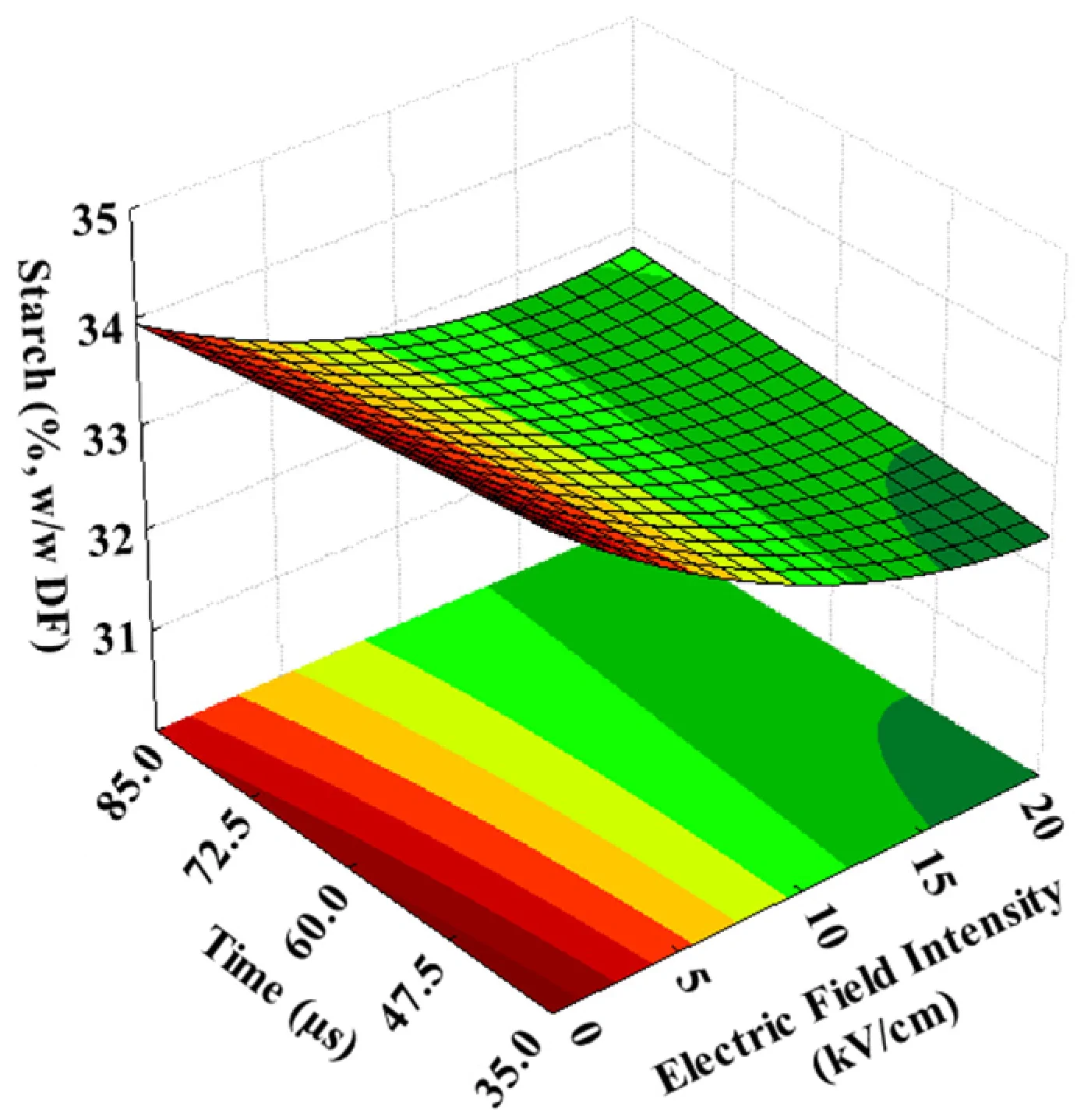
Response surface plots of the starch from *Q. robur* acorns obtained by pulsed electric field extraction.

The electric field intensity had a significant positive quadratic effect on starch yields, indicating that starch extraction yields decrease when lower to intermediate electric field intensities are used, but they increase when higher field intensities are applied (Table 1 and Figure 3).

One possible reason is that the applied electric field intensity was insufficient to disrupt the cell structure and liberate starch granules. According to the literature, the electric field intensity is the most important variable concerning PEF-assisted extraction, while extraction time plays a secondary role [31]. Thus, the processing conditions may not create enough transmembrane potential required to maximize starch yields [18].

### 2.2. Effect of Extraction Time

#### 2.2.1. Phenolic and Tannin Contents

Time had a significant negative quadratic effect on the extraction of phenolics and hydrolyzable tannins (Table 1). The response surface analysis (Figure 1A,B) showed that intermediate treatment times resulted in the highest recovery, while shorter or longer extraction times reduced the extraction. A similar effect was reported for potato peels, defatted flaxseed cake, and brewers’ spent grain [22,24,32]. In contrast to these results, time had a significant positive quadratic effect in the case of cocoa bean shell and barberry [23,33]. Increasing the extraction time initially enhanced the recovery of phenolics and tannins by increasing cell membrane permeabilization [28,34]. However, the recovery was negatively affected when higher times were applied, which is consistent with the observations reported for the extraction of phenolics from basil leaves [35]. This may indicate that higher extraction times may have led to compound degradation [28].

#### 2.2.2. Antioxidant Activity

Time also had a significant negative quadratic effect on the antioxidant activity (Table 1). The response surface analysis also indicated that the antioxidant activity reached a maximum at intermediate times before decreasing when longer extraction times were applied (Figure 2). This behavior followed that observed for phenolics and hydrolyzable tannins, indicating that both were contributors to the antioxidant activity. Since phenolic compounds are well established as antioxidants, the reduction in their recovery is in line with the observed reduction in antioxidant activity. The decrease in antioxidant activity after longer extraction times may be due to compound degradation released during PEF treatment responses [27].

#### 2.2.3. Starch Content

Time had no significant effect on the starch yields, showing that starch extraction is governed by the electric field intensity rather than time (Table 1 and Figure 3).

### 2.3. Models Fit, Optimization, and Validation

The statistical results indicate that the quadratic polynomial regression models are robust in predicting the impact of the electric field intensity and treatment time (Table 2). All models show a high statistical significance (*p* < 0.001), and the total contribution of the global models ranged from 90.85% for starch to 95.50% for the antioxidant activity. However, the fact that the lack of fit was significant in all response variables, even though the quadratic models were able to explain most variability, suggests that there must be additional complex interactions between the independent variables in the studied extractions.

Regarding phenolics and hydrolyzable tannins, the regression results indicate a similar behavior. The sequential sum of squares (SSS) attributes the highest value to the linear effect of the electric field intensity (2.94 for phenolics and 15.86 for the hydrolyzable tannins); the adjusted sum of squares (ASS) showed that the quadratic effect of the treatment time is the independent variable with the most impact on the extraction.

The electric field intensity had no significant contribution to phenolics, but the quadratic effect of this variable was highly significant for the hydrolyzable tannins. This indicates the existence of an inflection point among the different intensities applied. The additional strong quadratic effect of the treatment time confirms that the increase in treatment time does not necessarily increase the response variable, indicating possible compound degradation phenomena when longer treatment times are applied. The interaction between the linear effect of the electric field intensity and treatment time was not significant.

Concerning antioxidant activity, this response variable was found to be the most sensitive and complex of all response variables. The linear effect of the treatment time was the independent variable with the most contribution (62.29%). Unlike what was observed for phenolics and hydrolyzable tannins, the interaction between the electric field intensity and treatment time was significant on the antioxidant activity, retaining 16.46% of the contribution, showing a synergistic effect on the antioxidant activity.

Lastly, starch yields had a completely different statistical behavior compared to the previous response variables. Starch extraction was mainly governed by the linear effect of the electric field intensity, having a significant contribution of 75.20%. Since treatment time has no statistical relevance, this shows that starch extraction is mainly influenced by the quadratic effect of the electric field intensity, having an additional significant interaction between the electric field intensity and treatment time, which has a 4.57% contribution to the extraction.

The models used to describe the extraction of phenolics, tannins, starch, and antioxidant activity values had a good fit for the experimental data, as they presented high determination coefficients ($r^{2}$ > 0.90) and satisfactory goodness of fit with low mean errors (Table 3).

The adjusted coefficient values ($r_{adj}^{2}$ > 0.88) showed that most variation in the response was explained by the models. Moreover, the models are promising for predicting new responses due to the high predicted coefficients ($r_{pred}^{2}$ > 0.81).

Then, the individual mathematical models were optimized, and the optimum extraction conditions and the predicted values are displayed in Table 4. To achieve model validation, additional extractions and trials under these conditions were conducted. Validation was achieved since the difference between the predicted and experimental values was lower than 10% and the desirability values were also high.

Next, a multifactorial optimization was performed to maximize the starch yields and the extraction of phenolics and tannins with the highest antioxidant activity. The predicted optimum extraction condition was an electric field intensity of 0.1 kV/cm and a time of 63.3 µs with a desirability of 87%. Such a desirability value shows good agreement among the optimization objectives, demonstrating that this selected condition balances the extraction of starch, phenolic compounds and tannins.

To validate the predictive capacity of the multifactorial optimization, additional trials were also conducted under this condition. The experimental values were in good agreement with the predicted values (less than 10% difference), confirming the model’s adequacy (Table 5).

Since there were no appreciable differences between the observed values for the hydrolyzable tannins between the individual and the multifactorial optimization, and the starch yields obtained under multifactorial optimization were superior to those of the individual optimization, the multifactorial condition was selected for the next analysis.

### 2.4. Comparison with an Alkaline Extraction

To evaluate the potential of PEF as a sustainable alternative for acorn processing, the starch obtained under optimum conditions was compared with the starch obtained via alkaline extraction. The alkaline extraction had a significantly higher starch yield than PEF (Table 5). The higher extraction yields may be due to sodium hydroxide’s ability to remove proteins, lipids, and other non-starch constituents, as well as disrupt the cell membrane, easing the recovery of starch granules [14,15]. Despite the low starch yield, PEF has several important advantages over alkaline extraction. Firstly, only water was used as an extraction solvent, eliminating the need for chemical reagents and reducing the environmental concerns regarding wastewater generation and downstream washing steps. Secondly, it allows the simultaneous recovery of starch and tannin-rich extracts, which increases the overall valorization of *Q. robur* acorns. This approach is particularly attractive for a sustainable biorefinery strategy, where the main goal is to maximize the utilization of all components of a given biomass. A particularly noteworthy finding was the substantially higher recovery of hydrolyzable tannins obtained by PEF. Under multifactorial conditions, the hydrolyzable tannin recovery was 11 times higher than that via alkaline extraction. Tannins are high-value compounds that can be used as natural tanning agents in leather production, offering a better alternative to the currently used chromium salts [9,10,11,12,13]. As a result, although PEF yielded less starch, it created another added value stream that is not obtained via alkaline extraction. From an industrial standpoint, the choice between alkaline extraction and PEF depends on the final application. If the main objective is maximizing starch yields, then alkaline extraction is the most effective option. But when sustainability, clean-label processing, and simultaneous recovery of high-value compounds are considered, PEF extraction is the preferred alternative. With all points considered, PEF should not be seen simply as an alternative extraction technique for starch extraction but rather as an integrated extraction technique capable of generating more than one value-added stream from a single biomass while reducing the environmental impact associated with alkaline extraction.

### 2.5. SEM

**Figure 4:**
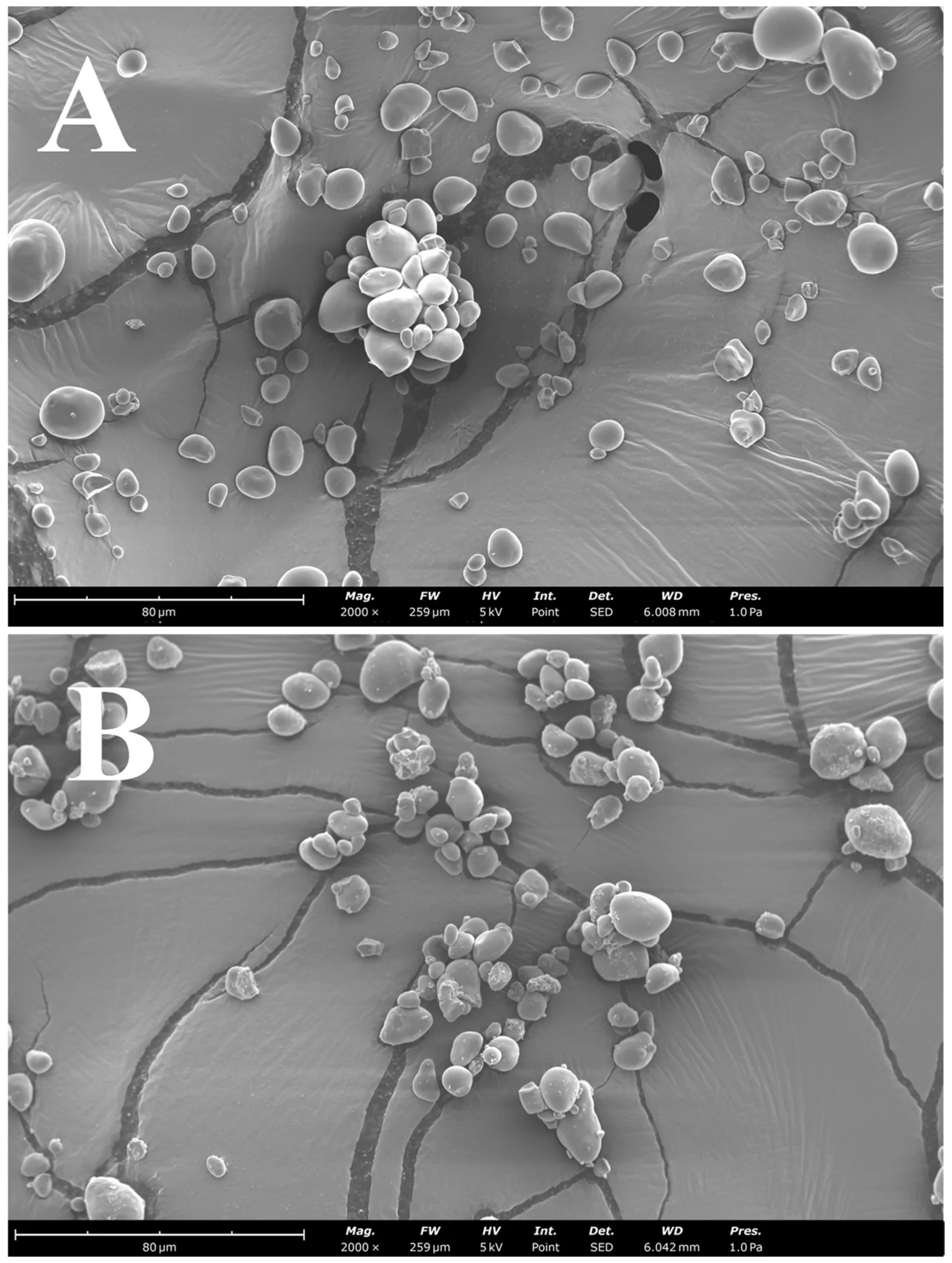
SEM micrographs of the isolated acorn starch granules by the (**A**) alkaline extraction and (**B**) PEF extraction under optimum conditions (0.1 kV/cm and 63.3 μs).

Scanning electron microscopy was used to evaluate the morphology of starch granules extracted under multifactorial conditions and to compare them with those obtained via alkaline extraction. Both extraction methods produced starch granules were mostly round, oval, and semi-circular in shape (Figure 4), as previously observed for *Q. robur* acorn starch [36]. Additionally, no evidence of granule rupture, surface erosion, or structural collapse was observed, indicating that both extraction methods preserve the overall starch granule integrity. The PEF-extracted starch granules had smooth surfaces and well-defined edges, showing that the optimum multifactorial conditions did not cause mechanical damage. This finding follows previous studies showing that low electric field intensities increase the permeability of cell membranes while preserving the native structure of starch granules [18,36]. The presence of small particles on the surface may be due to the presence of impurities that are retained during extraction and sieving [37,38]. Similarly, the starch obtained via alkaline extraction showed intact granules with similar morphology. Although alkaline extraction is more effective in removing protein, lipids, and other non-starch components from the granule surface, no major alterations in the granule shape were observed under these extraction conditions. This is in line with previous research [14,15]. Since no differences were observed between these two starches regarding external morphology, this indicates that the lower starch yields obtained under multifactorial conditions may not be related to structural damage to the starch granules but rather to differences in extraction efficiency. Thus, the lower starch yields obtained under multifactorial conditions are more likely related to an incomplete release of the starch granules from the acorn flour than to degradation of the starch granules. Overall, this analysis brought to light that PEF technology can be used to extract native starch without causing visible damage to the starch granules’ morphology. This also reinforces the potential of PEF as a green extraction technology capable of simultaneously producing clean starches while recovering tannin-rich extracts from the same acorn flour.

### 2.6. Gelatinization Properties

The gelatinization properties of the starch extracted under multifactorial conditions and the starch extracted via alkaline extraction were evaluated by measuring both solubility and swelling powers using temperatures ranging from 50 to 100 °C (Table 6). No solubility or swelling power was observed at 50 °C for either starch, indicating that this temperature is insufficient to trigger gelatinization. At this point, the crystalline regions of starch granules remain intact, restricting water absorption, and no amylose can leach [36]. As the temperature increased from 60 to 100 °C, both starches showed increases in solubility and swelling power values, highlighting the disruption of the hydrogen bonds between starch chains by the water molecules and, consequently, the loss of the crystalline regions during gelatinization.

Even though both starches had similar temperature-dependent behavior, the alkaline-extracted starch showed significantly higher solubility than the PEF starch extracted under multifactorial conditions. This greater solubility indicates increased amylose leaching during gelatinization, suggesting that alkaline extraction promoted changes that facilitated the diffusion of amylose molecules into the aqueous medium. One possible reason is that alkaline extraction might have disrupted other molecular associations involving amylose, lipids, and proteins with the starch granules. Their removal might have increased the mobility of amylose chains, which enhanced their release during gelatinization. In contrast, the PEF treatment appears to have had little to no influence on these molecular interactions, leading to lower amylose leaching and, consequently, to lower solubility. In contrast to solubility, no significant differences were observed among the studied starches over the temperature range. The swelling power primarily reflects the ability of amylopectin to absorb and retain water molecules [39]. Similar swelling behavior suggests that the amylopectin network remained intact by both extraction methods. Both swelling power and solubility are very important to understand the effect of an extraction process on the starch structure. The observed higher solubility values of the alkaline-extracted starch indicate greater mobility of the amylose chains during gelatinization, while the similar swelling power values between both starches indicate that the amylopectin architecture remained essentially intact. These results are in line with the SEM analysis, showing that the extraction methods did not lead to important morphological changes to the starch granules. Overall, these results show that PEF extraction conserves the native functional behavior of the acorn starch while avoiding structural modifications associated with alkaline extraction. These findings also support the potential of PEF as a technology to produce clean-label starches with properties fitted for food applications.

## 3. Conclusions

This research shows that PEF technology is an effective and environmentally friendly approach for the simultaneous extraction of starch, polyphenols and tannins with high antioxidant activity from *Q. robur* acorns using only water as an extraction solvent. The optimum extraction conditions were identified at an electric field intensity of 0.1 kV/cm for 63.3 µs, providing an overall desirability of 87%. This confirms the suitability of PEF to recover simultaneously more than one value-added component from acorns. Although PEF produced a lower starch yield compared to alkaline extraction, it achieved a higher recovery of hydrolyzable tannins while eliminating the need to use chemical reagents. The obtained extract represents a source of natural tannins for sustainable leather production, while the extracted starch is a clean-label ingredient obtained through a greener technology. Furthermore, both structural and functional characterization of the extracted starch shows that PEF was able to preserve the morphology of the native starch granules, as confirmed by SEM. Also, the similar swelling powers of both PEF-obtained starch and alkaline starch denote that the amylopectin framework was preserved by both extraction methodologies. Even though the alkaline-extracted starch had higher solubility, the PEF-extracted starch had its functional properties preserved while avoiding possible structural changes linked to alkaline extraction. Overall, these results show that PEF should be regarded as an integrated technology capable of producing simultaneously more than one high-value product from the same source. The combination of a clean-label produced starch and the obtention of a tannin-rich extract using only water as the extraction solvent, PEF stands as a more sustainable alternative for the valorization of underused acorns and supporting the transitions towards more circular and environmentally responsible food production systems. Future studies should be conducted to improve starch recovery while preserving the native starch functionality. Additionally, other important starch properties, such as rheological and thermal properties, should be investigated to provide a better platform for the identification of its applications.

## 4. Materials and Methods

### 4.1. Acorn Harvesting and Flour Preparation

*Q. robur* acorns were collected in Parque Nacional da Peneda-Gerês (Assento, Terras de Bouro, Braga, Portugal) on 18 November 2019. The *Q. robur* oaks were identified based on their anatomical features and leaf morphology. The most accessible and isolated trees were found at Parque Cerdeira (41°45′46.0″ N; 8°11′24.2″ W). Only mature acorns without putrefied tissues, mechanical damage, and/or spoilage by larvae were manually harvested from the ground beneath the oaks’ canopies [40]. Acorns were transported to the laboratory, cleaned with tap water and gently dried with a fabric cloth, and stored at −20 °C until further use.

For acorn flour preparation, the shells were removed by hand using a knife, and the cotyledons were ground and sieved through a 1.0 mm and a 0.5 sieve mesh. Acorn flour had a moisture content of 57.1% according to AOAC 925.10. The produced flour was homogenized, packed under 75% vacuum, and stored at −20 °C until further use.

### 4.2. Alkaline Extraction of Starch and Phenolics

The alkaline extraction procedure was adapted from that reported with minor modifications [14]. An 8 g portion of acorn flour was mixed with 100 mL of 0.25% NaOH (*w*/*v*) under constant agitation for 24 h at 5 °C. The suspension was centrifuged for 20 min at 3000× *g*, and the supernatants were stored at −20 °C. The supernatant was analyzed for total phenolic content, hydrolyzable tannins, condensable tannins, and its antioxidant activity was measured. The obtained residue was sieved through 160, 80, and 40 μm diameter mesh sieves with the aid of water and left to stand overnight at 4 °C to collect the starch granules liberated during PEF treatment. The water was discarded, and the starchy material was dried in a ventilated drying chamber at 45 °C. Extractions were performed in triplicate.

### 4.3. PEF Extraction

#### 4.3.1. Experimental Design

A Box–Behnken experimental design was employed to evaluate the effect of the electric field intensity and treatment time (Table 7). The experiments were performed in a randomized order to minimize systematic experimental bias. The best extraction conditions were then determined by analyzing the data using a response surface approach [41]. Error assessment was based on three replications of the central point (E10/t60).

#### 4.3.2. Extraction of Starch and Phenolics

Extractions were conducted in an industrial bench-scale continuous PEF system (Diversified Technologies, Bedford, MA, USA). The acorn flour suspensions (8%, *w*/*v*) were prepared with water and pumped at 800 mL/min through the treatment chamber, which consisted of two ceramic parallel electrodes (3.5 mm diameter and 4.7 mm gap). The suspension was at 22 °C and had an initial conductivity of 1094 ± 7 µS/cm before treatment. Exponential unipolar pulses with a pulse width of 9.3 µs were applied at 1400 Hz. The difference treatment times were calculated according to the literature by multiplying the pulse width by 3 pulse levels, that is, 3.76, 6.45, and 9.14 pulses, which corresponds to 35, 60, and 85 µs [42].

After treatment, the electrical conductivity increased no more than 8%, while the temperature of the suspension remained below 26 °C. Suspensions were centrifuged at 3000× *g* for 20 min and the supernatants were stored at −20 °C. The supernatant was analyzed for total phenolic content, hydrolyzable tannins, condensable tannins, and its antioxidant activity was measured. The obtained residue was screened using 180 and 40 μm diameter mesh sieves, thoroughly washed with water and left to stand overnight at 4 °C to collect the starch granules liberated during PEF treatment. The starchy material was collected and dried at 45 °C in a ventilated drying chamber. Extractions were performed in triplicate. All observed results are summarized in Table 8.

#### 4.3.3. Optimization and Validation

The observed data were fitted to a second-order polynomial model (Equation (1)), where *Y* is the predicted response, *β*_0_ is the model intercept coefficient, *β_t_*, and *β_E_* are the linear coefficients, *β_tt_* and *β_EE_* are the quadratic coefficients, *β_tE_* is the linear interactive coefficients for the independent variables time (*t*) and electric field intensity (*E*), respectively.

(1) $$Y\;\left(t,E\right)=\;\beta_{0}+\;\beta_{t}t\;{+\beta }_{E}E+\;\beta_{tt}t^{2}\;{+\beta }_{EE}E^{2}\;{+\beta }_{tE}tE$$

The regression coefficients were estimated by analysis of the variance and used to obtain the optimum extraction conditions and desirability values [41]. The goodness of fit and additional extractions was performed under the optimum predicted conditions to validate the models.

### 4.4. Analytical Determinations

#### 4.4.1. Starch Yields

The starch yields were determined according to Equation (2), where *m* is the mass of dried starchy material and *m_f_* the mass of weighted fresh flour on a dry basis [43].

(2) $$Yield\;\left(\%\right)=\frac{m}{m_{f}}\times 100$$

Results were expressed as grams of starchy material per 100 g of dried flour on a dry basis (%, w/w DF).

#### 4.4.2. Scanning Electron Microscopy

A Phenom XL G2 scanning electron microscope (Thermo Fischer Scientific, Eindhoven, The Netherlands) was used to analyze the starch granules’ morphology. The starch samples were fastened on observation pegs coated with double-sided carbon tape (NEM tape, Nisshin, Japan) and coated with gold using a sputter coater (Polaron, Watford, Hertfordshire, UK). Samples were inspected using a working distance of 6.0 mm, a field width of 259 µm, and a magnification of 2000×. When the apparatus was running at a high vacuum (1.0 Pa) and an accelerating voltage of 5 kV, the secondary electron detector was utilized for SEM experiments. Granules morphology was evaluated according to reported descriptors [36].

#### 4.4.3. Phenolic Content

The phenolic content was determined using the Folin-Ciocalteu method [44]. A 30 µL aliquot of extract was mixed with 100 µL of Folin-Ciocalteu (20%, *v*/*v*) and with 100 µL of Na_2_CO_3_ (7.4%, *w*/*v*). After allowing it to react for 1 h in the dark at room temperature, the absorbance was recorded at 750 nm using an Agilent BioTek Synergy H1 multidetector plate reader (Winooski, VT, USA) and the Agilent BioTek Gen5 software (version 3.04.17) (Winooski, VT, USA). Quantification was performed in triplicate, and results were expressed as milligrams of gallic acid equivalent per gram of fresh flour on a dry flour (mg GA Eq./g DF).

#### 4.4.4. Condensed and Hydrolyzable Tannin Contents

Condensed tannin contents were measured using the vanillin method [44]. A 50 µL aliquot of extract was mixed with 150 µL of vanillin reagent (1% *w*/*v* in 7 M H_2_SO_4_) reacting for 15 min in the dark at room temperature. Absorbance was read at 500 nm using a multidetector plate reader with the Gen5 software. Quantification was performed in triplicate, and results were expressed as milligrams of catechin equivalent per gram of dried flour (mg CA Eq./g DF).

Hydrolyzable tannin contents were measured using the potassium iodate method [44]. A 40 µL aliquot of extract was mixed with 200 µL of KIO_3_ (2.5%, *w*/*v*) and orbital shaken (205 cpm) for 20 s. Maximum absorbance was recorded at 550 nm at room temperature using a multidetector plate reader with the Gen5 software. Quantification was performed in triplicate, and results were expressed as milligrams of tannic acid equivalent per gram of dried flour (mg TA Eq./g DF).

#### 4.4.5. Antioxidant Activity

Antioxidant activity was measured by the ABTS scavenging activity method using a 96-well microplate [44]. A 20 µL aliquot of extract was mixed with 180 µL of ABTS^•+^ reagent for 5 min at room temperature in the dark. Absorbance was recorded at 734 nm using a multidetector plate reader with the Gen5 software. The ABTS^•+^ reagent was prepared by mixing 10 mL of ABTS solution (7 mM) with 10 mL K_2_S_2_O_8_ (2.44 mM) for 16 h, filtered using a 0.45 µm syringe filter, and diluted with water to an absorbance of 0.70 ± 0.02. Quantification was performed in triplicate, and results were expressed as milligrams of Trolox equivalent per gram of dried flour (mg TX Eq./g DF).

#### 4.4.6. Gelatinization Properties

A water bath was used to incubate a starch suspension in water (1%, *w*/*v*) using temperatures ranging from 50 to 100 °C for 30 min. After cooling, suspensions were centrifuged for 30 min at 4100× *g* [36]. While the gels were weighed exactly, the supernatants were freeze-dried using a Biobase Model BK-FD12P (Biobase Group, Jinan, China). Solubility and swelling power were computed according to Equations (3) and (4), respectively where $W_{LS}$ is the weight of the lyophilized supernatant (g), $W_{S}$ is the starch weight in dry basis (g), and $W_{G}$ is the gel weight (g).

(3) $$S\;(\%)=\frac{W_{LS}}{W_{s}}\times 100\;$$

(4) $$SP\;\left(\frac{g}{g}\right)=\frac{W_{G}}{W_{s}\;(1-\frac{S}{100})}\;$$

Solubility and swelling power results were expressed in percentage (%; gram of solubilized starch per 100 g of starchy material on a dry basis) and in gram of gel per g of non-solubilized starchy material on a dry basis (g/g), respectively.

### 4.5. Statistical Analysis

Statistical analyses were performed using a one-way ANOVA, followed by the Tukey’s HSD test for multiple comparisons at 5% significance level and the response surface models were obtained using the STATISTICA 7 software. All results were presented as the mean ± standard deviation.

## Figures and Tables

**Table 1:** Regression coefficients of the models of phenolics (mg GA Eq./g DF), hydrolyzable tannins (mg TA Eq./g DF), ABTS antioxidant activity (mg TX Eq./g DF), and starch content (%; w/w DF) for *Q. robur* acorns. Significant correlations are written in bold.

Regression Coefficient ^a,b^	Phenolics	Hydrolyzable Tannins	ABTS	Starch
$\beta_{0}$	**23.00500**	**50.34000**	**51.26300**	**34.72600**
$\beta_{E}$	−0.01730	0.02570	**0.29180**	**−0.235300**
$\beta_{EE}$	−0.00108	**−0.01022**	**−0.00574**	**0.004710**
$\beta_{t}$	**0.10560**	**0.29180**	**0.20820**	−0.005800
$\beta_{tt}$	**−0.00092**	**−0.00275**	**−0.00099**	−0.000040
$\beta_{tE}$	−0.00018	0.00108	**−0.00347**	**0.000984**

GA: Gallic acid; TA: Tannic acid; TX: Trolox; DF: Dried flour; (^a^) The coefficients *β_0_*, *β_E_*, *β_E_*, *β_t_*, *β_tt_*, and *β_tE_* are expressed as mg GA, TA, or TX Eq./g DF; mg GA, TA, or TX Eq./g DF. kV. cm^−1^; mg GA, TA, or TX Eq./g DF. kV^2^. cm^−2^; mg GA, TA, or TX Eq./g DF. µs; mg GA, TA, or TX Eq./g DF. µs^2^; and mg GA, TA, or TX Eq./g DF. µs. kV. cm^−1^ for phenolics, hydrolyzable tannins, and ABTS antioxidant activity models, respectively; (^b^) The coefficients *β_0_*, *β_E_*, *β_E_*, *β_t_*, *β_tt_*, and *β_tE_* are expressed as g/100 g DF; g/100 g DF. kV. cm^−1^; g/100 g DF. kV^2^. cm^−2^; g/100 g DF. µs; g/100 g DF. µs^2^; and g/100 g DF. µs. kV. cm^−1^ for starch models, respectively.

**Table 2:** Complete ANOVA regression analysis of the quadratic polynomial equation models for phenolics, hydrolyzable tannins, ABTS antioxidant activity, and starch content from *Q. robur* acorns.

Response	Regression Source	DF	SSS	Contribution	ASS	AMS	F-Value	*p*-Value
Phenolics	Global model	5	5.30	92.01%	5.30	1.06	36.86	0.000
	E (kV/cm)	1	2.94	51.06%	0.02	0.02	0.64	0.435
	t (µs)	1	0.30	5.29%	1.46	1.46	50.97	0.000
	E (kV/cm) × E (kV/cm)	1	0.37	6.40%	0.06	0.06	2.01	0.175
	t (µs) × t (µs)	1	1.67	28.99%	1.67	1.67	58.06	0.000
	E (kV/cm) × t (µs)	1	0.02	0.27%	0.02	0.02	0.55	0.471
	Error	16	0.46	7.99%	0.46	0.03		
	Lack-of-fit	3	0.32	5.57%	0.32	0.11	9.97	0.001
	Pure error	13	0.14	2.42%	0.14	0.01		
	Total	21	5.76	100%				
Hydrolysable tannins	Global model	5	48.92	93.29%	48.92	9.79	44.47	0.000
	E (kV/cm)	1	15.86	30.25%	0.04	0.04	0.18	0.673
	t (µs)	1	5.72	10.90%	11.18	11.18	50.82	0.000
	E (kV/cm) × E (kV/cm)	1	11.79	22.48%	5.19	5.19	23.57	0.000
	t (µs) × t (µs)	1	14.98	28.57%	14.98	14.98	68.08	0.000
	E (kV/cm) × t (µs)	1	0.57	1.09%	0.57	0.57	2.60	0.126
	Error	16	3.52	6.71%	3.52	0.22		
	Lack-of-fit	3	3.01	5.73%	3.01	1.00	25.37	0.000
	Pure error	13	0.51	0.98%	0.51	0.04		
	Total	21	52.45	100%				
ABTS antioxidant activity	Global model	5	34.57	95.50%	34.57	6.91	67.97	0.000
	E (kV/cm)	1	1.21	3.33%	5.24	5.24	51.48	0.000
	t (µs)	1	22.55	62.29%	5.69	5.69	55.96	0.000
	E (kV/cm) × E (kV/cm)	1	2.93	8.09%	1.64	1.64	16.08	0.001
	t (µs) × t (µs)	1	1.93	5.33%	1.93	1.93	18.98	0.000
	E (kV/cm) × t (µs)	1	5.96	16.46%	5.96	5.96	58.59	0.000
	Error	16	1.63	4.50%	1.63	0.10		
	Lack-of-fit	3	1.29	3.58%	1.29	0.43	16.87	0.000
	Pure error	13	0.33	0.92%	0.33	0.03		
	Total	21	36.20	100%				
Starch	Global model	5	9.52	90.86%	9.52	1.90	31.79	0.000
	E (kV/cm)	1	7.88	75.20%	3.40	3.40	56.84	0.000
	t (µs)	1	0.00	0.04%	0.00	0.00	0.07	0.788
	E (kV/cm) × E (kV/cm)	1	1.15	11.01%	1.10	1.10	18.42	0.001
	t (µs) × t (µs)	1	0.00	0.03%	0.00	0.00	0.05	0.822
	E (kV/cm) × t (µs)	1	0.48	4.57%	0.48	0.48	8.00	0.012
	Error	16	0.96	9.14%	0.96	0.06		
	Lack-of-fit	3	0.88	8.42%	0.88	0.29	50.34	0.000
	Pure error	13	0.08	0.72%	0.08	0.01		
	Total	21	10.47	100%				

SSS: Sequential sum of squares; ASS: Adjusted sum of squares; AMS: Adjusted mean squares.

**Table 3:** Goodness of fit of the quadratic polynomial equation models for phenolics, hydrolyzable tannins, ABTS antioxidant activity, and starch content from *Q. robur* acorns.

Parameter	Phenolics	Hydrolysable Tannins	ABTS	Starch
$r^{2}$	0.9201	0.9329	0.9550	0.9086
$r_{adj}^{2}$	0.8952	0.9119	0.9410	0.8800
$r_{pred}^{2}$	0.8476	0.8741	0.9056	0.8136
MSE	0.2457	0.1600	0.0740	0.0435
MAE	0.4166	0.3555	0.2339	0.1715
MRQE	0.0004	0.0001	0.0000	0.0000
MRAE	0.0166	0.0063	0.0039	0.0052

MSE: Mean squared error; MAE: Mean absolute error; MRSE: Mean relative squared error; MRAE: Mean relative absolute error.

**Table 4:** Individual optimization of phenolics (mg GA Eq./g DF), hydrolyzable tannins (mg TA Eq./g DF), ABTS antioxidant activity (mg TX Eq./g DF), and starch content (%; w/w DF) from *Q. robur* acorns.

Response Variable	Optimum Condition	Desirability	Predicted		Observed Value
			Value	Prediction Interval (95% Confidence)	
Phenolics	E0.1/t57.7	0.9813	26.038	25.686; 26.441	26.477 ± 0.142
Hydrolysable tannins	E4.1/t53.7	0.9539	58.244	57.189; 59.299	57.103 ± 1.205
ABTS	E0.1/t85.0	1.0000	61.821	61.022; 62.620	63.827 ± 0.983
Starch	E0.1/t35.0	1.0000	34.453	33.840; 35.066	31.022 ± 0.520

GA: Gallic acid; TA: Tannic acid; TX: Trolox; DF: Dried flour.

**Table 5:** Multifactorial optimization of phenolics (mg GA Eq./g DF), hydrolyzable tannins (mg TA Eq./g DF), ABTS antioxidant activity (mg TX Eq./g DF), and starch content (%; w/w DF) from *Q. robur* acorns.

Response Variable	Predicted		Observed Value (E0.1/t63.3)	Alkaline
	Value	Prediction Interval (95% Confidence)		
Phenolics (mg GA Eq./g DF)	26.008	25.605; 26.490	27.799 ± 1.171 ^b^	22.433 ± 0.052 ^a^
Hydrolyzable tannins (mg TA Eq./g DF)	57.794	56.681; 58.908	56.690 ± 0.580 ^b^	5.097 ± 0.027 ^a^
ABTS (mg TX Eq./g DF)	60.488	59.731; 61.245	59.127 ± 1.850 ^a^	51.312 ± 0.000 ^a^
Starch (%, w/w DF)	34.180	33.599; 34.761	34.514 ± 0.449 ^a^	48.974 ± 1.220 ^b^

GA: Gallic acid; TA: Tannic acid; TX: Trolox; DF: Dried flour. The differences between the observed value, control, and alkaline were represented by lower-case letters, and values in the same row with the same letters are not significant (*p* > 0.05).

**Table 6:** Solubility and swelling power values of *Q. robur* acorn starches extracted by PEF under optimum conditions and the corresponding alkaline counterparts.

Parameter	T (°C)	K0.1/t63.3	RALK
Solubility (%)	50	n.d.	n.d.
	60	2.03 ± 0.23 ^aA^	4.35 ± 0.09 ^bA^
	70	5.73 ± 0.08 ^aB^	8.71 ± 0.13 ^bB^
	80	9.71 ± 0.24 ^aC^	12.32 ± 0.72 ^bC^
	90	15.00 ± 0.68 ^aD^	19.60 ± 1.23 ^bD^
	100	17.28 ± 0.76 ^aE^	22.41 ± 0.92 ^bD^
Swelling power (g/g)	50	n.d.	n.d.
	60	5.60 ± 0.20 ^aA^	6.07 ± 0.17 ^aA^
	70	7.95 ± 0.06 ^aB^	7.95 ± 0.24 ^aB^
	80	9.57 ± 0.72 ^aC^	10.12 ± 0.24 ^aC^
	90	11.16 ± 0.28 ^aD^	11.70 ± 0.12 ^aD^
	100	13.05 ± 0.03 ^aE^	13.17 ± 0.35 ^aE^

n.d.: not detected; K0.1/t63.3: *Q. robur* acorn starch extracted under optimum conditions by PEF (0.1 kV/cm for 63.3 µs); RALK: *Q. robur* acorn starch extracted via alkaline. Significant differences between temperatures are represented by capital-case letters, and values in the same column with the same letters are not significant (*p* > 0.05). Significant differences between starches are represented by lower-case letters, and values in the same row with the same letters are not significant (*p* > 0.05).

**Table 7:** Experimental conditions for PEF extraction with the two independent variables (electric field intensity and extraction time).

Experiment	Experimental Design		Extraction Condition
	Electric Field Intensity (E; kV/cm)	Treatment Time (t; µs)	
1	0.1	35	E0.1/t35
2	0.1	60	E0.1/t60
3	0.1	85	E0.1/t85
4	10	35	E10/t35
5	10	60	E10/t60
6	10	85	E10/t85
7	20	35	E20/t35
8	20	60	E20/t60
9	20	85	E20/t85

**Table 8:** Observed data of phenolics (mg GA Eq./g DF), hydrolyzable tannins (mg TA Eq./g DF), condensed tannins (mg CA Eq./g DF), ABTS antioxidant activity (mg TX Eq./g DF), and starch yields (%; w/w DF) from *Q. robur* acorns.

Extraction Condition	Phenolics	Hydrolyzable Tannins	Condensed Tannins	ABTS	Starch
E0.1/t35	25.54 ± 0.03	57.37 ± 0.04	n.d.	57.33 ± 0.12	34.21 ± 1.10
E0.1/t60	25.99 ± 0.10	57.61 ± 0.23	n.d.	60.57 ± 0.12	34.34 ± 1.23
E0.1/t85	25.42 ± 0.04	55.43 ± 0.35	n.d.	61.48 ± 0.20	34.04 ± 0.79
E10/t35	25.22 ± 0.02	56.29 ± 0.11	n.d.	58.18 ± 0.05	33.39 ± 1.26
E10/t60	25.66 ± 0.01	58.26 ± 0.29	n.d.	60.47 ± 0.07	32.92 ± 1.20
E10/t85	24.68 ± 0.10	54.96 ± 0.18	n.d.	61.55 ± 0.19	32.66 ± 0.27
E20/t35	24.73 ± 0.11	54.68 ± 0.01	n.d.	58.77 ± 0.21	32.12 ± 0.56
E20/t60	24.83 ± 0.22	55.04 ± 0.09	n.d.	59.26 ± 0.13	32.69 ± 1.24
E20/t85	24.43 ± 0.09	53.81 ± 0.26	n.d.	59.47 ± 0.21	32.92 ± 0.67

n.d.: not detected (LOQ: <1.25 µg/mL); GA: Gallic acid; TA: Tannic acid; CA: Catechin; TX: Trolox; DF: Dried flour.

## Data Availability

The data presented in this study are openly available in article.
